# ZOOM or Non-ZOOM? Assessing Spinal Cord Diffusion Tensor Imaging Protocols for Multi-Centre Studies

**DOI:** 10.1371/journal.pone.0155557

**Published:** 2016-05-12

**Authors:** Rebecca S. Samson, Simon Lévy, Torben Schneider, Alex K. Smith, Seth A. Smith, Julien Cohen-Adad, Claudia A. M. Gandini Wheeler-Kingshott

**Affiliations:** 1 NMR Research Unit, Queen Square MS Centre, Department of Neuroinflammation, UCL Institute of Neurology, London, United Kingdom; 2 Institute of Biomedical Engineering, Ecole Polytechnique de Montreal, Montreal, QC, Canada; 3 Functional Neuroimaging Unit, University of Montreal, Montreal, QC, Canada; 4 Philips Healthcare, Guilford, Surrey, United Kingdom; 5 Vanderbilt University Institute of Imaging Science (VUIIS), Vanderbilt University, Nashville, Tennessee, United States of America; 6 Department of Biomedical Engineering, Vanderbilt University, Nashville, Tennessee, United States of America; 7 Department of Radiology and Radiological Sciences, Vanderbilt University, Nashville, Tennessee, United States of America; 8 Brain MRI 3T Center, C. Mondino National Neurological Institute, Pavia, Italy; 9 Department of Brain and Behavioural Sciences, University of Pavia, Pavia, Italy; University Medical Center Utrecht, NETHERLANDS

## Abstract

The purpose of this study was to develop and evaluate two spinal cord (SC) diffusion tensor imaging (DTI) protocols, implemented at multiple sites (using scanners from two different manufacturers), one available on any clinical scanner, and one using more advanced options currently available in the research setting, and to use an automated processing method for unbiased quantification. DTI parameters are sensitive to changes in the diseased SC. However, imaging the cord can be technically challenging due to various factors including its small size, patient-related and physiological motion, and field inhomogeneities. Rapid acquisition sequences such as Echo Planar Imaging (EPI) are desirable but may suffer from image distortions. We present a multi-centre comparison of two acquisition protocols implemented on scanners from two different vendors (Siemens and Philips), one using a reduced field-of-view (rFOV) EPI sequence, and one only using options available on standard clinical scanners such as outer volume suppression (OVS). Automatic analysis was performed with the Spinal Cord Toolbox for unbiased and reproducible quantification of DTI metrics in the white matter. Images acquired using the rFOV sequence appear less distorted than those acquired using OVS alone. SC DTI parameter values obtained using both sequences at all sites were consistent with previous measurements made at 3T. For the same scanner manufacturer, DTI parameter inter-site SDs were smaller for the rFOV sequence compared to the OVS sequence. The higher inter-site reproducibility (for the same manufacturer and acquisition details, i.e. ZOOM data acquired at the two Philips sites) of rFOV compared to the OVS sequence supports the idea that making research options such as rFOV more widely available would improve accuracy of measurements obtained in multi-centre clinical trials. Future multi-centre studies should also aim to match the rFOV technique and signal-to-noise ratios in all sequences from different manufacturers/sites in order to avoid any bias in measured DTI parameters and ensure similar sensitivity to pathological changes.

## 1. Introduction

The spinal cord (SC) is a common site of involvement in neurological disorders such as multiple sclerosis, amyotrophic lateral sclerosis, spinal cord injury and neuromyelitis optica [1], and high field post mortem magnetic resonance imaging (MRI) studies have confirmed both focal and diffuse abnormalities in these conditions [2–6]. Diffusion Tensor Imaging (DTI) provides quantitative information about the microstructure of tissue *in vivo* and the diffusion of water molecules can be altered in pathology. By modelling the signal behaviour of water diffusion in tissue using DTI, it is possible to derive several indices that may be sensitive biomarkers for characterising tissue microstructural abnormalities [7]. These include fractional anisotropy (FA), mean diffusivity (MD), and axial and radial diffusivities (AD and RD), all of which have previously been shown to be affected by various pathological tissue changes such as demyelination, axonal damage and inflammation. Consequently, spinal cord DTI has the potential to provide sensitive biomarkers that could be used in future clinical trials of potential therapies for neurodegenerative diseases.

However, there are many technical challenges associated with making quantitative measurements in the SC *in vivo* due to its small cross-sectional size and the potential for SC motion (both physiological and involuntary patient motion) during scans [8, 9]. The most commonly used pulse sequence for diffusion-weighted MRI is echo planar imaging (EPI), because it is very rapid, and gives high signal-to-noise ratio (SNR) efficiency (i.e. SNR per square root of the acquisition time). However, various issues may arise with the use of EPI readout, in particular geometric distortions due to susceptibility differences of air, bone and tissue, and through-plane dephasing resulting in signal drop-out [10].

The small physical dimensions of the cord contribute to partial volume effects with surrounding CSF, which may also result in errors in measured diffusion parameters and constrains the actual pixel size, especially in the plane orthogonal to the main axis of the spinal cord [11, 12].

Additionally, susceptibility-induced distortions become more of a concern at higher image resolution. In fact, the FOV in the phase encoding direction per each echo-train readout and the time between the acquisition of two consecutive k-space lines both affect the bandwidth (which is proportional to 1/echo spacing), and, therefore, the magnitude of image distortions.

Physiological motion in particular may bias MD measurements [13] and result in ghosting artefacts [14]. The spinal cord moves within the spinal canal, simultaneously with CSF flow, and is also close to the heart and lungs, which undergo periodic motion. In addition, respiratory-related shifts in the B_0_ field induce dynamic distortions along the phase-encoding direction [15]. It is possible to use cardiac and respiratory gating to synchronise the acquisition of data with the cardiac and respiratory cycles, however, these methods may result in potentially large increases in acquisition time.

Many artefacts in EPI result from the much smaller bandwidth per pixel (frequency difference between adjacent voxels) along the phase encoding (PE) direction compared with the frequency encoding axis. This means that chemically shifted signals from fat will be displaced by many pixels, and that small magnetic field differences will result in much greater geometric distortions along the PE axis, for example due to susceptibility differences of air, bone and soft tissue. Acquiring data oriented transverse to the cord may provide better image quality than sagittal or coronal images, as there will then be less main magnetic field (B_0_) variation across the slice, especially if aligned with the centre of the intervertebral discs or of the vertebral bodies [16].

There is a trade-off between high spatial resolution and adequate SNR due to the small size of the cord; for reliable depiction of anatomical detail an in-plane resolution of at least 1x1mm^2^ is typically required, which again provides a good argument for acquiring data in an axial orientation (thereby resulting in the highest spatial resolution in the axial plane).

In order to minimize image distortions, the PE FOV per echo-train readout may be reduced, for example, by using parallel imaging, thereby reducing the acquisition window duration and hence image distortions. However, this may not be available along all imaging axes, depending on the type of receiver coil used [17]. Ramp sampling and a higher receiver bandwidth can also be used to reduce the echo spacing. The bandwidth in EPI is often set to the maximum possible (at a cost of a reduction in SNR) to minimize the pervasive distortions resulting from EPI-based DTI [18]. Alternatively the pixel resolution could also be reduced; however this is not a desirable option for the spinal cord, in order to avoid partial volume effects. For small objects such as the cord, the echo train length (ETL) could be reduced by reducing the FOV, which may result in aliasing (wrap-around artefact), unless reduced FOV (rFOV) techniques are employed [19–25]. However, these techniques are not standardly available on all clinical scanners, and may require a research agreement with the manufacturer and/or even sequence programming efforts.

Here we aimed to develop two different SC DTI protocols for use in multi-centre studies, one using research options to enable the use of rFOV techniques, and one using only standard options available on clinical scanners, but neither requiring pulse sequence programming. Below we give a brief introductory description of the techniques used in this study.

### 1.1. Reduced FOV (rFOV) sequence

For the first protocol we aimed to use a rFOV sequence (an adaptation of the zonally magnified oblique multi-slice (ZOOM) [26] sequence for Philips sites and single shot 2 dimensional radio frequency (2DRF) selective excitation [23] for Siemens. The rFOV EPI sequence (ZOOM or 2DRF excitation) makes use of a greatly reduced FOV (either through an inner volume imaging technique or a reduced excitation area) to achieve a shorter ETL, thereby reducing artefacts caused by susceptibility changes between soft tissue and the adjacent vertebrae.

Reduced FOV methods are often used in SC imaging to limit the extent of coverage in the PE direction, thereby reducing the required number of k-space lines, which in turn reduces off-resonance induced artefacts (at the cost of an SNR reduction the size of the square root of the PE FOV reduction factor).

#### 1.1.1. ZOOM-EPI sequence: Description of technique

The ZOOM sequence has seen several implementations, all based on slightly different combinations of imaging gradients, and excitation and refocusing pulses. All ZOOM methods exploit the concept of inner volume imaging [25], and enable the selective excitation and refocusing of a narrow FOV, whilst avoiding signal originating from outside the desired FOV. The method therefore enables the measurement of diffusion parameters with reduced image distortions [20, 22, 26, 27]. The pulse sequence used for the present method consists of a slice-selective 90° pulse tilted at a small angle (11.8° for the implementation described below; see also [27]) with respect to the imaging plane (to enable multiple-slice acquisitions with no slice gap), followed by a non-coplanar 180° refocusing pulse, such as in [20]. This results in only a narrow region of the volume at the intersection being both excited and refocused. In addition this implementation uses special optimized outer volume suppression pulses with very sharp transition profiles adjacent to the FOV in the PE direction to eliminate aliasing from extraneous signal outside the encoding area, arising from imperfect RF pulses [27]. For DTI, diffusion-sensitizing gradients are introduced on either side of the refocusing pulse of the ZOOM-EPI sequence.

#### 1.1.2. 2DRF excitation: Description of technique

Instead of a 1D excitation pulse, 2D spatially selective RF pulses can be used to excite only a small FOV. These 2D-RF pulses have excitation profiles which are periodic in either the phase or the slice direction, followed by a 180° refocusing pulse which is used to refocus only the main lobe of the excitation.

The 2DRF excitation excites a stripe of magnetization within the object, with the size of the stripe selected in such a way that it covers the desired FOV. The desired FOV is also oversampled to avoid aliasing of the transition region where the magnetization fades out.

One possible disadvantage of 2DRF excitation, however, is that the 2DRF pulses may have long durations, causing the slice profiles to be sensitive to off resonance effects and lengthening the minimum echo time (TE) achievable, with consequences on the data SNR. Here, we used the implementation proposed in [23].

### 1.2. Outer volume suppression (OVS)

The second method we chose to examine is a basic DTI sequence with outer volume suppression (OVS). This method uses OVS pulses prior to the excitation pulse, and can be implemented by positioning suppression ‘bands’ anteriorly and posteriorly (A/P) to the spinal cord (or in general, in any PE direction) to avoid aliasing. This option is available on most commercial MRI scanners, and is often already utilized with other spinal cord anatomical sequences.

We therefore aimed to develop two SC DTI protocols (neither requiring any pulse sequence programming): one which can be implemented on any standard clinical MRI scanner (i.e. OVS), and one utilising research options that are either not yet commercially available or that have just been released onto the market as specific packages, which therefore are not necessarily available clinically (i.e. ZOOM or 2DRF), the goal being to ascertain which protocol could enter into clinical trials. The sequences were tested on three scanners at three different sites (one in Europe, one in the US and one in Canada) from two different manufacturers by quantitative assessment of MD, FA, AD (principal eigenvalue of the diffusion tensor), and RD (average of second and third eigenvalues) in five healthy subjects at each site. Intra- and inter-site reproducibilities were calculated for each sequence.

## 2. Materials and Methods

### 2.1. MRI Acquisition

Data were acquired on 3T scanners at three sites (1 (London)–Philips Achieva (Philips Healthcare, Best, The Netherlands), 2 (Vanderbilt)–Philips Achieva (Philips Healthcare, Best, The Netherlands), and 3 (Montreal)–TIM Trio (Siemens Healthcare, Erlangen, Germany)). The two Philips systems are equipped with 80 mT/m gradients with maximum 200 mT/m/ms slew rate and a 16-channel neurovascular receive coil. The Siemens system is equipped with 40 mT/m gradients with maximum 200 mT/m/ms slew rate and 12-channel head + 4-channel neck receive-only coils. This study was approved by the local ethics committee at each of the three sites (London ethics board: London-Harrow; Vanderbilt ethics board: Health Sciences Subcommittee of the Vanderbilt University Institutional Review Board; Montreal ethics board: Comité mixte d’éthique de la recherche du Regroupement Neuro-Imagerie Québec (RNQ)) and all subjects provided informed consent in writing prior to the commencement of data acquisition. Five subjects were scanned at each of the 3 different sites (in total, 8M, 7F, mean age 26.9 ± 5.6 years).

Where possible, protocols for the two different scanner manufacturers utilised identical acquisition parameters, though some options required manufacturer-specific parameters.

Site-independent identical parameters: 21 axial slices of 5mm thickness were acquired (with phase encoding anterior/posterior (A/P)) using a single-shot EPI readout, with in-plane resolution 1x1mm^2^, and the FOV centred at the level of the C3-4 intervertebral disc, spanning levels C1-C6 in all volunteers. The imaging plane was prescribed to be perpendicular to the cord at the C3-4 level. Cardiac gating via peripheral pulse oximetry was used in all protocols to reduce physiological motion. The diffusion weighting (b-value) was set to 750s/mm^2^ for all sequences. For the OVS sequences, saturation bands were positioned anteriorly and posteriorly to avoid aliasing. All other (manufacturer-specific) sequence details are given in Table 1.

**Table 1:** Manufacturer-specific acquisition details.

Acquisition parameter	Philips-specific OVS	Siemens-specific OVS	Philips-specific ZOOM	Siemens-specific 2DRF
TR (ms)*	~7000	~7000	~7000	~7000
**TE (ms)**	72	89	50	81
**Matrix size**	100x100	100x100	64x48	64x38
**Bandwidth (Hz)**	893.1	1085	893.1	1100
**SENSE (Philips) or GRAPPA (Siemens) acceleration factor**	1.5	2.0	1.5	2.0
**Partial Fourier?**	No	6/8	No	7/8
**Number of diffusion-weighted directions**	32	30	32	30
**Number of b = 0 acquisitions**	4	4	4	4

*Times approximate due to cardiac gating

Additionally, standard clinical T_2_-weighted anatomical data were acquired at all sites to enable template-based analysis of diffusion-weighted data, in a two-step process with co-registration to the template performed via the anatomical images.

For the two Philips sites a 3D Volume Isotropic Turbo Spin-echo Acquisition (VISTA) sequence was used, with the following acquisition parameters: TR = 2000ms, TE = 120ms, sagittal acquisition, FOV = 250x250x60mm^3^, with isotropic 1x1x1mm^3^ resolution, no SENSE, halfscan = 0.64, bandwidth = 408.5Hz/voxel, and NSA = 1.

At the Siemens site (site 3), the equivalent sequence Sampling Perfection with Application optimized Contrasts using different flip angle Evolution (SPACE) was used, with a slab-selective fast spin echo (TR = 1500 ms, TE = 119 ms, sagittal acquisition, FOV = 384x384x52m^3^, with isotropic 1x1x1mm^3^ resolution, flip angle = 120°, bandwidth = 723 Hz/voxel).

### 2.2. Image analysis

#### 2.2.1. Semi-automatic ASM whole cord region-of-interest (ROI) analysis

Standard DTI parameters were analysed by a single observer (RS) by fitting the data to a diffusion tensor model using the open-source Camino toolkit [28] (using linear regression to compute a least squares fit of the diffusion tensor to logarithmic measurements). Mean b = 0 images were used for segmentation of the cord from surrounding CSF, using an active surface model (ASM) [29] implemented in Jim (Xinapse systems, www.xinapse.com), with manual adjustments to the whole cord regions of interest (ROIs) made where necessary. The ROI is traced as a continuous line and voxels are included in the final mask if at least 50% of them belong to the inner part of the ROI. These were applied to parameter maps to give whole cord (C1-C6) mean DTI parameter values for each subject and protocol.

#### 2.2.2. Template-based analysis

Additionally, all data were processed using the Spinal Cord Toolbox (SCT, http://sourceforge.net/projects/spinalcordtoolbox/ [30]) to show the feasibility of performing fully automated template-based analysis, thereby ensuring reproducible results without user-bias during ROI selection. DWI data were motion-corrected using the method of Xu *et al*. [31] as implemented in the SCT. Then, DWI data were registered to the MNI-Poly-AMU spinal cord template [32], using a series of affine and diffeomorphic transformations between the mean of the b = 0 data, the T_2_-weighted anatomical data and the MNI-Poly-AMU template. The diffusion tensor was then computed using SCT (using DIPY (http://nipy.org/dipy)), and DTI parameters were estimated in the spinal cord [33] using a maximum a posteriori method. This method enables biases related to partial volume effects to be overcome [33].

#### 2.2.3. Statistical analysis

Intra- and inter-site comparisons of whole-cord MD, FA, AD and RD values obtained in native space (using the semi-automatic ASM method in Jim for segmentation, with adjustments if needed) were performed. Paired t-tests were used to look for differences between parameter values obtained with different protocols at the same site, and one-way ANOVA tests were used to examine differences between the 3 sites, for both the OVS and ZOOM protocols. All statistical analyses were performed for both semi-automatic whole cord ROI- and template-based data. Coefficients of Variation (CVs; calculated using CV = 100*(mean/SD) %) were used to assess reproducibility both within and between sites. Statistical analysis was performed using SPSS version 21.0 (SPSS, Inc., Chicago, IL, USA).

#### 2.2.4. SNR measurements in b = 0 images

Average SNR measurements for each site were measured via the “difference method” [34], using the whole cord ROI masks (generated using the ASM implemented using Jim [29]) with manual editing if needed) in two b = 0 images for each subject) and are given in Table 2.

**Table 2:** Average SNR values for each site and protocol, calculated from two b = 0 images for each subject, using the “difference method” [34] in whole cord ROI masks.

Site/protocol	Average b = 0 SNR value
1—OVS	6.74 (± 3.71)
2 –OVS	9.79 (± 3.88)
3 –OVS	7.21 (± 1.34)
1—ZOOM	7.71 (± 4.22)
2—ZOOM	10.9 (± 0.62)
3—2DRF	6.94 (± 1.34)

## 3. Results

### 3.1. MRI acquisition

**Fig 1:**
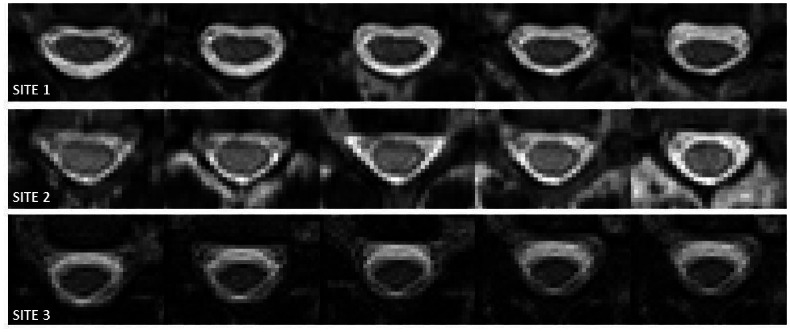
Anatomical images centred at levels C3/C4, similarly to the images shown in Figs 2 and 3, for an example single subject (not the same subject at each of the three sites) acquired at each of the 3 sites (London, Vanderbilt and Montreal; 1–3 from top to bottom, respectively).

**Fig 2:**
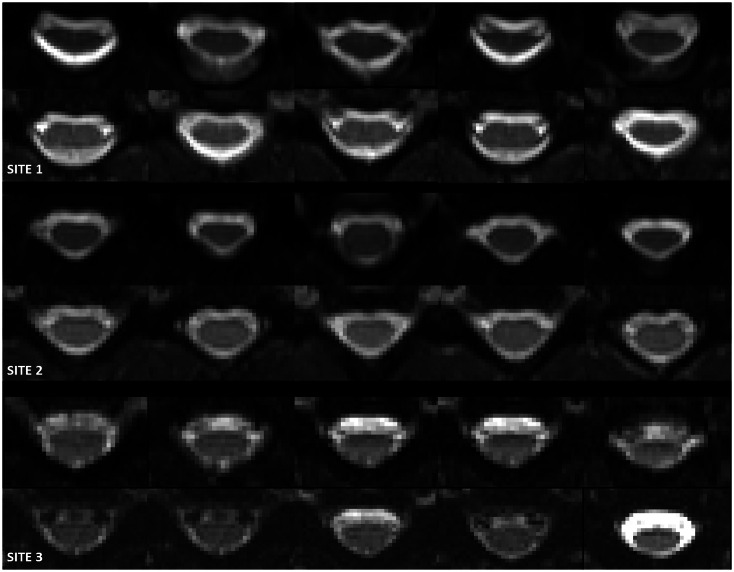
Central 5 slices of mean b_0_ images for an example single subject (not the same subject at each of the three sites) for the OVS protocol (top), followed by the rFOV protocol (bottom), acquired at each of the 3 sites (London, Vanderbilt and Montreal; 1–3 from top to bottom, respectively).

**Fig 3:**
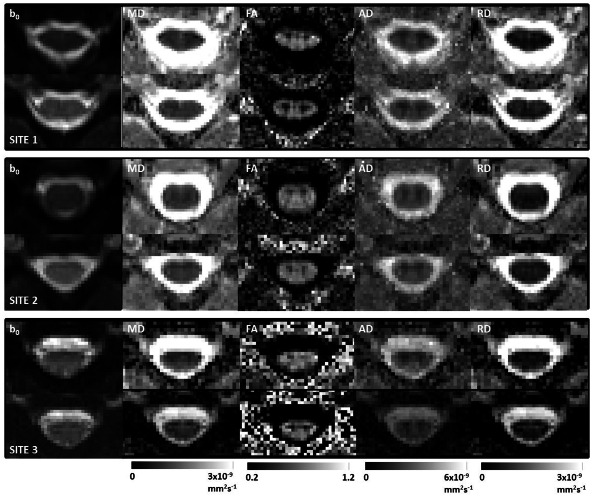
Single subject central slice parameter maps for both protocols acquired at sites 1, 2 and 3 (London, Vanderbilt and Montreal) respectively from top to bottom (for the same subjects as in Figs 1 and 2, but as before not the same subject at each fo the three sites). For each figure the images from left to right are: mean b_0_, MD, FA, AD and RD, with images acquired using the OVS protocol on top and the rFOV implementation at the bottom, and associated colour bars given underneath.

Fig 1 shows 5 slices of anatomical images centred at levels C3/C4, similarly to the images shown in Figs 2 and 3, for a single subject acquired at each of the 3 sites (London, Vanderbilt and Montreal; 1–3 from top to bottom, respectively).

Fig 2 shows the central 5 slices of mean b = 0 images for a single subject for the OVS protocol (top), followed by the rFOV protocol (bottom), acquired at each of the 3 sites (1–3 from top to bottom). Images acquired using the rFOV sequence (bottom row) appear less distorted than those acquired using the OVS sequence (top row) for both sites. It can also be seen that the 2DRF excitation images acquired at site 3 have a reduced SNR compared to the ZOOM images acquired at sites 1 and 2 (see also Table 2). Additionally, there are some variations in contrast observable, in particular in the CSF.

Fig 3 shows single subject DTI-derived indices at a central slice for both protocols at each site 1, 2 and 3 respectively from top to bottom. Note Fig 3 shows data derived from the same subjects as in Figs 1 and 2. For each figure the images from left to right are: mean b = 0, MD, FA, AD and RD, with images acquired using the OVS sequence implementation on top and the rFOV implementation at the bottom, as well as associated colour bars. As discussed previously, at all sites the rFOV images appear less distorted than those acquired using the OVS sequence. For site 3 the parameter maps obtained using the 2DRF sequence are a little noisier than those obtained using the ZOOM sequence (see Fig 3).

**Fig 4:**
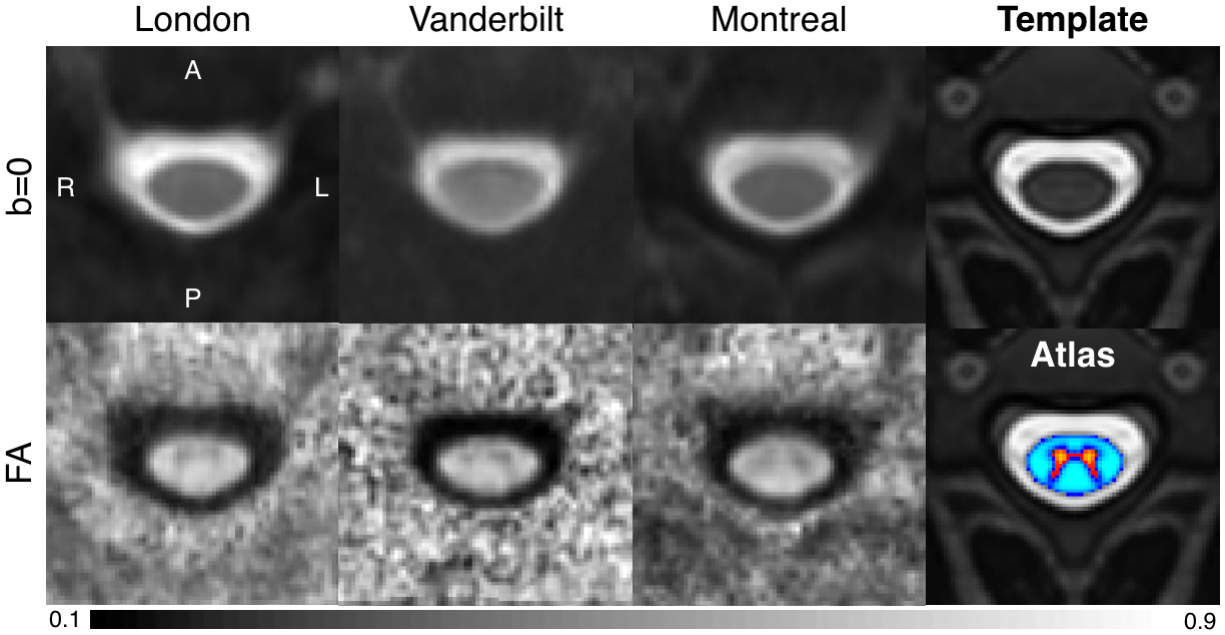
Results of registration to the MNI-Poly-AMU template and group averaging (N = 5; different subjects were scanned at each site) for sites 1 (London), 2 (Vanderbilt) and 3 (Montreal) (b = 0 images are shown in the top row, and FA maps in the bottom). The 4^th^ column shows the T_2_-weighted template (top) and the white/grey matter probabilistic atlas (bottom).

Fig 4 shows b = 0 images and FA maps obtained from analysis of the data acquired using the rFOV sequence at each site (averaged across subjects), after registration with the MNI-Poly-AMU template. The fourth column shows the T_2_-weighted template (top) and the white/grey matter probabilistic atlas (bottom). The white matter atlas was used here to compute DTI metrics while allowing the possibility of taking into account potential partial volume effects (as opposed to the semi-automatic ROI analysis, where partial volume effects are accounted for only by inclusion/exclusion of voxels based on their % involvement in the cord ROI).

### 3.2. Image analysis

#### 3.2.1. Semi-automatic ASM whole cord region-of-interest (ROI) analysis

Mean MD, FA, AD and RD values (± standard deviations (SDs)) for each site and protocol (using both the semi-automatic binary ROI analysis, and automatic template-based analysis with SCT [30], accounting for partial volume effect (with subscript ‘sct’)) are given in Table 3.

**Table 3:** Cervical cord (C1-C6) mean MD, FA, AD and RD values (± SD) for each site and protocol, given both for the manual ROI analysis performed in native space and also using automatic analysis with the SCT (with subscript ‘sct’).

	MD (x 10^−6^ mm^2^s^-1^)	MD_sct_ (x 10^−6^ mm^2^s^-1^)	FA	FA_sct_	AD (x 10^−6^ mm^2^s^-1^)	AD_sct_ (x 10^−6^ mm^2^s^-1^)	RD (x 10^−6^ mm^2^s^-1^)	RD_sct_ (x 10^−6^ mm^2^s^-1^)
**1—OVS**	1.29 (±0.21)	1.28 (±0.22)	0.59 (±0.06)	0.59 (±0.03)	2.21 (±0.22)	2.22 (±0.29)	0.84 (±0.21)	0.82 (±0.19)
**2—OVS**	1.21 (±0.13)	1.13 (±0.11)	0.60 (±0.07)	0.63 (±0.06)	2.08 (±0.09)	2.07 (±0.08)	0.77 (±0.17)	0.66 (±0.14)
**3—OVS**	1.10 (±0.06)	1.00 (±0.09)	0.63 (±0.03)	0.64 (±0.04)	1.95 (±0.07)	1.77 (±0.23)	0.68 (±0.06)	0.56 (±0.10)
**1—ZOOM**	1.17 (±0.05)	0.96 (±0.08)	0.59 (±0.02)	0.65 (±0.02)	2.09 (±0.12)	1.86 (±0.17)	0.71 (±0.07)	0.52 (±0.03)
**2—ZOOM**	1.16 (±0.13)	1.08 (±0.08)	0.62 (±0.05)	0.61 (±0.03)	2.03 (±0.12)	1.98 (±0.09)	0.73 (±0.15)	0.62 (±0.09)
**3—2DRF**	0.93 (±0.20)	0.75 (±0.09)	0.60 (±0.05)	0.66 (±0.03)	1.55 (±0.27)	1.43 (±0.18)	0.63 (±0.17)	0.46 (±0.15)

Intra-site SDs varied for all parameters measured using the two sequences at each site; In London (site 1) parameters obtained using ZOOM had lower SDs, Vanderbilt (site 2) had similar parameter SDs for both sequences, and Montreal (site 3) had lower DTI parameter SDs for the OVS sequence than the 2DRF sequence.

Site 3 (Montreal) shows a small difference in measured AD (p = 0.018) between the OVS and 2DRF sequences, but no other significant differences in measured parameters between sequences were observed. No significant differences in any DTI parameter were observed between measurements made using the two protocols on either of sites 1 and 2.

Intra-vendor coefficients of variation (CVs (%)), measured considering only site 1 and 2, for MD, FA, AD and RD were 1.20, 0.61, 2.08 and 0.76 for OVS and 1.05, 0.59, 1.81 and 0.67 for ZOOM. Inter-site CVs measured considering all three sites, for MD, FA, AD and RD were 7.99, 3.01, 6.06 and 10.6 respectively for the OVS sequence and 12.3, 2.70, 15.4 and 8.11 for the ZOOM sequence.

No significant differences between DTI parameters measured using the OVS sequence were observed (tested using a one-way ANOVA). However, significant differences between site 3 (2DRF sequence) and the 2 other sites (ZOOM sequence) were observed as follows:

MD was found to be significantly different in site 3 from site 1 (p = 0.01) and site 2 (p = 0.018), and AD was found to be significantly different in site 3 from site 1 (p<0.001) and site 2 (p = 0.001).

#### 3.2.2. Template-based analysis

Similarly to the semi-automatic ROI analysis, intra-site SDs varied for all parameters measured using the two sequences at each site; In London (site 1) parameters obtained using ZOOM had lower SDs, Vanderbilt (site 2) had mostly lower parameter SDs for the ZOOM than the OVS sequence, and Montreal (site 3) had mostly lower DTI parameter SDs for the OVS sequence than the 2DRF sequence.

However, almost all parameter values were found to be significantly different when measured via OVS compared to ZOOM/2DRF (tested using paired t-tests), with the exception of the MD and FA at site 3 (Montreal) and FA at site 2 (Vanderbilt).

Intra-vendor coefficients of variation (CVs (%)), measured considering only site 1 and 2, for MD, FA, AD and RD were 9.53, 4.57, 5.24 and 16.1 for OVS and 9.13, 3.98, 5.40 and 14.8 for ZOOM. Inter-site CVs measured considering all three sites, for MD, FA, AD and RD were 12.7, 4.59, 11.4 and 18.9 respectively for the OVS sequence and 17.8, 4.01, 16.4, and 15.4 for the ZOOM sequence.

Comparing the three sites using a one-way ANOVA, for the OVS sequence significant differences were observed for MD between sites 1 and 3 (p = 0.013), for RD between sites 1 and 3 (p = 0.018) and for AD between sites 1 and 3 (p = 0.007) and 2 and 3 (p = 0.05). For the rFOV sequence, significant differences were observed for MD (site 1 vs 3 (p = 0.002), and 2 vs 3 (p = 0.023)), FA (site 2 vs 3 (p = 0.023)), RD (site 2 vs 3 (p = 0.026)) and AD (site 1 vs 3 (p = 0.001), site 2 vs 3 (p<0.001)).

#### 3.2.3. SNR measurements in b = 0 images

Average (± SD) SNR measurements for each site (measured via the “difference method” [34], using whole cord ROI masks in two b = 0 images for each subject) are given in Table 2.

## 4. Discussion

We have presented a multi-centre comparison of two SC DTI acquisition protocols, one which can be implemented on any standard clinical MRI scanner (OVS), and one utilising research options not yet commercially available (reduced FOV technique).

SC MD, FA, RD and AD values obtained using both acquisition protocols at all sites were consistent with previous measurements made at 3T [35–37].

Some variations in contrast were observable in b = 0 images, in particular in the CSF for site 3, which are likely due to some remaining CSF pulsation effects. Cardiac gating implementations are different between the Siemens site and the Philips. It is possible that susceptibility artefacts close to vertebral discs may also have caused some signal dropouts.

Lower CVs were demonstrated for rFOV than for OVS when only considering results from the two Philips sites (i.e. the ZOOM sequence only), via semi-automatic ROI analysis, and to a lesser extent via template-based analysis. However, some differences between measured DTI parameters were observed at site 3, using the 2DRF sequence compared to the ZOOM sequence implemented at the other two sites.

It should, however, be acknowledged that the two implementations of the rFOV protocol are very different; for instance, the Siemens protocol has a longer TE (mainly due to the 40mT/m gradient strength vs. 80mT/m on the Philips) which has an impact on SNR, in addition to the use of a 2DRF pulse on the Siemens Trio system, and this lower SNR can in turn can affect the accuracy of the fitted DTI parameters, as well as possibly affecting the exact water compartment under investigation. Future work is also warranted to assess other acquisition issues such as shimming and cardiac gating for each vendor and each sequence implementation. Encouragingly, for the two Philips sites the intra-vendor CVs were reduced to extremely low values (all <2.5%) (and similar to those observed in a previous multi-centre diffusion MRI study in the brain [38]), with lower CVs for the ZOOM sequence than the OVS sequence, also considering that the groups of subjects were different, although of similar age and gender distribution.

Unfortunately identical rFOV methods were not available on the scanners from both manufacturers used in this study. Since the goal of this study was to evaluate sequences not requiring any pulse programming (one standard sequence, and one available with research options), for potential future application in multi-centre trials, we did not compare additional in-house methods in order to match sequences more closely from both vendors.

Standard deviations in SNR measurements across the 5 subjects were found to be large, in particular for site 1. It was not possible to measure SNR using measurements of signal and noise e.g. in a corner of the image, because of inhomogeneity correction algorithms used, and the fact that there are typically no regions of “air/no signal” in rFOV images. Therefore, it was necessary to use the “difference method”, with SNR values measured over the whole cord, meaning that it is possible that errors due to motion may have occurred and affected SNR measurements in all subjects. The SNR measured for the 2DRF sequence at site 3 was lower than for the OVS sequence at that site, whereas it was found to be higher for the ZOOM sequence than the OVS sequence for the 2 Philips sites, and the rFOV SNR was also lowest for site 3, which may be another explanation for the variability in measured DTI parameters observed between sites. As mentioned previously, the lower SNR at site 3 is mainly due to the lower maximum gradient strength on the Siemens Trio system, imposing a longer TE (81ms vs. 50ms for the Philips).

The Philips scanner software used in this study (release 3.2.1) does not allow the possibility of interleaving b = 0 images within the diffusion-weighted images without the use of pulse programming, which may have led to less reliable motion correction of our data. This feature will be included in future releases, which may even further improve the reproducibility of the measured DTI indices on this system, by enabling more robust motion correction [37, 39], and may in turn enable column- and tissue-specific quantification of DTI parameters.

Additionally the ongoing development of a ZOOM EPI method for the Siemens scanner as well as the latest improvements on gradient strength (80mT/m for the Prisma scanner) may improve inter-scanner reproducibilities of measured DTI parameters.

We also performed automatic template-based analysis of our data using SCT [30], which has the potential to enable a higher level of standardisation of spinal cord DTI, by providing a generic reference space (template and atlas), removing user bias thanks to the automatic ROI definition. Counterintuitively, inter-site CVs were found to be larger than those measured via semi-automatic ASM ROI analysis of the same subjects, and significant differences in parameter values were observed using the OVS vs. the rFOV sequence at each site, and between sites using the proposed protocol. The higher variability of template-based analysis is explained by a number of potential differences between analysis methods as raw data was processed with different pipelines including motion correction, diffusion tensor fitting algorithm, spinal cord ROI extraction and partial volume correction. It is important to underline that the current experimental design faces intrinsic intra-site and inter-site variabilities, reproducing the scenario of clinical trials or multi-center studies where different populations are studied at different sites. The intra-site variability comes from the inherent variability across subjects, while the inter-site variability comes from differences across scanners and MR procedure (subject positioning, coaching, slice orientation, etc.). These variabilities were more apparent using the template-based vs. semi-automatic ASM method. Given the differences in all processing steps, the source of variability is difficult to pin down without further analysis and the possible use of synthetic data where the ground truth is known a priori. Although synthetic data was already proposed in [33] to assess partial volume effects in quantifying spinal cord metrics with the SCT, designing in silico phantoms, replicating raw diffusion weighted data, with Rician noise characteristics typical of the low SNR spinal cord images, simulating also different amounts of motion, is warranted in order to develop and test the full image analysis pipeline.

In particular the two methods of analysis deal with partial volume effects very differently, where the SCT tries to estimate partial volume, while the ASM method simply excludes voxels that contribute less than 50% to the outlined spinal cord ROI and all included voxels are averaged with the same weights. This difference means that the intrinsic inter-subject and inter-scanner variability could be affected by a different handling of partial volume with the surrounding CSF [33], potentially masking real data variability. A question is whether SCT is therefore able to better capture differences in whole cord parameters across sites and across subjects, or whether these could be related to pre-processing steps of the data. Further investigations, including test-retest experiments to measure the “true” reproducibility, should be conducted, in order to further advance spinal cord multi-centre studies using such automatic methods.

We have demonstrated the feasibility of performing multi-centre SC DTI studies, but with the caveat that it is very important to try to match the sequence design as far as possible between sites, and in particular to ensure that sequences from different manufacturers/sites are SNR-matched, in order to avoid any bias in measured DTI parameters. The availability of different rFOV methods on different scanners was already a stepping stone for pushing spinal cord DTI towards clinical use, although the chapter has just been opened and requires international and joint efforts to speed up results.

A larger multi-centre study including both scan-rescan tests, and possibly scanning of the same subjects at different sites is desirable to further evaluate the protocols developed in terms of precision of the measurements and sensitivity to changes.

The higher inter-site reproducibility (for the same manufacturer and acquisition details, i.e. ZOOM data acquired at the two Philips sites) of the rFOV sequence compared to the OVS sequence supports the suggestion that making research options such as rFOV more widely available on clinical systems may improve accuracy of measurements obtained in multi-centre clinical trials. This would enable optimal protocols to be used in such studies, and allow multiple sites and manufacturers to match different sequences more closely, both in terms of implementation, and SNR.

## Supporting Information

S1 Dataset All measurements obtained for both the manual ROI-based analysis and the template-based analysis. (XLSX)
